# The Michigan University and Homœopathy

**Published:** 1876-06

**Authors:** 


The Michigan University and Homœopathy.—Since the organization of the School of Homœopathy in connection with the medical department of the University of Michigan—thus blending the two, and making it a copartnership business—the profession has been greatly disturbed, not only in the State where this monstrosity was brought forth, but over the entire country, as is evinced by the sharp discussions that have been going on for the past ten months.

From recent developments the faculty will be forced to recede from their position, as was predicted by the tone of the profession months ago.

At a recent meeting of the Michigan State Medical Society the action of the faculty was condemned, and a resolution introduced which refused admittance to the recent graduates of the University. The following is the part of the resolution excluding the graduates since the amalgamation: * * "*Provided*, That no person shall be admitted to membership who practices or professes to practice in accordance with any so-called "party," or sectarian school of medicine, or who has recently graduated from a school whose professors teach or assist in teaching those who propose to graduate in or practice irregular medicine." This resolution would have passed by a large majority, but, as it was an amendment to the constitution, it will lie over one year.

The Convention of Colleges recently in session, if we are correctly informed, condemned the faculty, or the amalgamation, in courteous but decided terms.

At the recent meeting of the American Medical Association, that body gave expression upon the subject in the following resolution: "*Resolved*, That members of the medical profession who in any way aid or abet the graduation of medical students in irregular or exclusive systems of medicine, are deemed thereby to violate the spirit of the ethics of the American Medical Association." It is evident that the voice of the profession is in decided opposition to the innovation being practiced by the Michigan University. The very best thing they can do will be to recede gracefully from their position.

